# HIV prevention for the next decade: Appropriate, person-centred, prioritised, effective, combination prevention

**DOI:** 10.1371/journal.pmed.1004102

**Published:** 2022-09-26

**Authors:** Peter Godfrey-Faussett, Luisa Frescura, Quarraisha Abdool Karim, Michaela Clayton, Peter D. Ghys, (on behalf of the 2025 prevention targets working group)

**Affiliations:** 1 Data for Impact Practice, UNAIDS, Geneva, Switzerland; 2 London School of Hygiene and Tropical Medicine, London, United Kingdom; 3 CAPRISA, Durban, South Africa; 4 Independent Consultant, Windhoek, Namibia

## Abstract

- UNAIDS and a broad range of partners have collaborated to establish a new set of HIV prevention targets to be achieved by 2025 as an intermediate step towards the sustainable development target for 2030.
- The number of new HIV infections in the world continues to decline, in part due to the extraordinary expansion of effective HIV treatment. However, the decline is geographically heterogeneous, with some regions reporting a rise in incidence. The incidence target that was agreed for 2020 has been missed.
- A range of exciting new HIV prevention technologies have become available or are in the pipeline but will only have an impact if they are accessible and affordable and delivered within systems that take full account of the social and political context in which most infections occur. Most new infections occur in populations that are marginalised or discriminated against due to structural, legal, and cultural barriers.
- The new targets imply a new approach to HIV prevention that emphasises appropriate, person-centred, prioritised, effective, combination HIV prevention within a framework that reduces existing barriers to services and acknowledges heterogeneity, autonomy, and choice.
- These targets have consequences for people working in HIV programmes both for delivery and for monitoring and evaluation, for health planners setting local and national priorities, and for funders both domestic and global. Most importantly, they have consequences for people who are at risk of HIV exposure and infection.
- Achieving these targets will have a huge impact on the future of the HIV epidemic and put us back on track towards ending AIDS as a public health threat by 2030.

## Abstract 2

Peter Godfrey-faussett and colleagues discuss approaches and considerations for HIV prevention in the next decade.

## Author summary

The world (through the United Nations General Assembly) has committed to ending AIDS as a public health threat by 2030. However, progress has not been sufficient and in 2020 there were over 1.5 million new HIV infections, compared to the target of 500,000 that had been adopted. The United Nations Joint Programme on AIDS therefore convened a broad range of partners to develop new targets for 2025 that will put us back on track to meet the 2030 goal. These targets are at the heart of the global HIV strategy for 2021 to 2025 and include targets to maximise HIV services, to minimise societal barriers, and to integrate HIV services within the wider health system.

This paper forms part of a larger PLoS Collection that describes the overall 2025 Targets process and lays out the exciting developments and prospects for HIV prevention and explains the rationale and the details of the new prevention targets.

The specific prevention targets signal a shift in the global HIV prevention strategy to maximise agency, equity, and efficiency. They aim to achieve a world where 95% of people who are exposed to HIV use “Appropriate, Person-centred, Prioritised, Effective, Combination Prevention”.

## Introduction

### Course correction needed to reach the 2030 target for the sustainable development goal for HIV

A major course correction is needed to maximise the chance of reaching the targets already established for 2030 in the Sustainable Development Goals (SDGs), which include “Ending the epidemic of AIDS by 2030” [1]. UNAIDS and partners from a diverse range of stakeholders have worked to elaborate a new set of guiding principles and to establish a new set of intermediate targets for 2025 taking into account recent advances in biomedical, social, and epidemiological sciences [2]. These deliberations and the targets that emerged from them are described in more detail in several papers in this Collection and are central to the current UNAIDS Global strategy [3].

This policy forum lays out the overarching prevention target, the principles for guiding prevention programmes, and highlights the ways in which these principles are incorporated into the mathematical models of the potential impact of implementing the 2025 global targets for the HIV response.

## Accomplishments of HIV prevention to date and remaining challenges

### Milestones for 2020 were missed

Global progress in controlling the HIV epidemic has been too slow. The target for 2020 included in the SDGs of 500,000 new infections has been missed [4]. There were 1.5 million new HIV infections in 2020 [5]. Although there is good evidence that people on effective treatment do not transmit HIV [6,7], scaling up treatment has not been sufficient to reduce the number of new HIV infections to the levels called for in successive political declarations. Primary prevention of new adult infections remains critical.

### HIV prevention has changed

The opportunities for more effective prevention of new HIV infections have expanded over the past decade. Oral pre-exposure prophylaxis (PrEP) has been demonstrated to be highly efficacious and often effective in public health practice [8–10]. Two monthly injections of a long-acting formulation of cabotegravir have been demonstrated in clinical trials to be more effective than oral PrEP [11,12]. Intra-vaginal silicone rings delivering dapivirine topically have also been shown to be moderately effective [13,14] and are included in the WHO HIV prevention guidelines as an additional prevention technology.

While new technologies are needed, their impact will be limited if real challenges in delivery such as barriers to access to services, cost, supply chain and logistics, monitoring, and evaluation are not addressed.

Developments in phylogenetics, mathematical modelling, and enhanced epidemiological surveillance are providing insights into the dynamics of transmission and acquisition of HIV infection related to age and gender [15]; increasing the geographic resolution [16]; and predicting current and future contributions to new infections of people with acute and early infection, those PLHIV who have not been previously diagnosed, those PLHIV who are not on treatment, those PLHIV whose virus is resistant to treatment, and those PLHIV who are no longer taking effective treatment [17].

### New HIV targets adopted for 2025

The UNAIDS 2020 “90-90-90” target has been a driving force behind the scale-up of effective HIV treatment, thus reducing onward transmission of HIV [18]. The implementation of HIV treatment services needs to address all the complexity of the diversity of the 37.7 million people estimated to be living with HIV in 2020 [19]. However, the success of the 90-90-90 treatment target arises both from the immediacy of saving lives by treating people living with HIV but also from the conceptual simplicity that every one of the 37.7 million people living with HIV needs to be diagnosed and linked to highly effective treatment. In contrast, HIV prevention is much more complex and the billions of people who are sexually active, use intravenous drugs or are born to mothers living with HIV require a mosaic of interventions and approaches [20]. This leads to a segmentation of the population into multiple overlapping groups according to age, gender, behaviour, sexual orientation and gender identity, geography, and social circumstance. Given the increasing number of prevention technologies and options alongside the many intersections with societal and legal barriers, the number of potential targets expands dramatically, and it is not possible to define a simple target with the specificity of 90-90-90.

**Fig 1:**
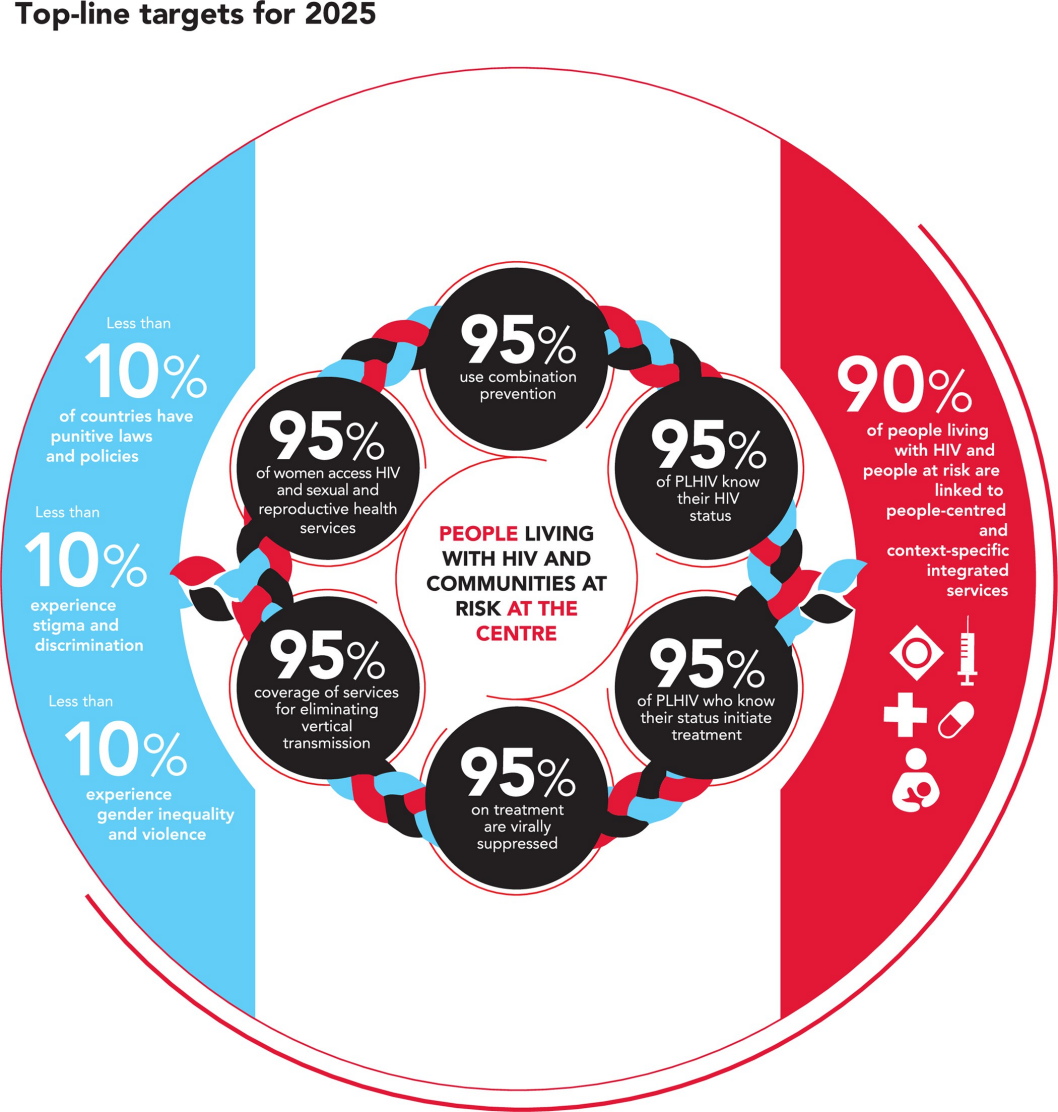
The UNAIDS 2025 Targets for the HIV response. The global targets for the HIV response, accepted by the UN, to be reached by 2025 include HIV services, Societal enablers, and Integration elements.

The 2025 Targets approach, shown diagrammatically in Fig 1, aims to reduce the separation between treatment and prevention and therefore has a set of 6 overarching targets for HIV services that are all set to an ambitious 95% [2,3].

## The new target and approaches for HIV prevention

### 95% of people at risk of HIV infection use appropriate, person-centred, prioritised, and effective, combination prevention options

The new overarching 95% prevention target is framed to maximise equity and impact. Sufficient access to prevention technologies and services is currently often inadequate even for established modalities such as condom provision or circumcision services [21] and does not meet the demands of populations to be served [22]. Increasing demand for services is most likely to arise from community-led approaches to planning, delivery, and monitoring in partnership with traditional health service providers [23].

The overarching target for prevention therefore refers to a proportion of all people at any risk of HIV being reached by effective, evidence-based services, rather than by utilisation of a specific tool.

For large populations in much of the world, the background rates of HIV and the behavioural choices of individuals means that the risk of acquiring HIV is minimal. For such people, the target can be met simply by ensuring a comprehensive understanding of sexual and reproductive health. However, as risks of HIV acquisition increase, a wider range of more HIV-specific tools will be needed and human and financial resources will need to be increased to meet the target.

However, overall resources for HIV have not increased in past years [5]. Increasing the total budget needs to be matched by ensuring fairness through innovative approaches to reduce costs of commodities and their delivery and through appropriate cost sharing internationally and locally [24]. Many sexual and reproductive health services and approaches that reduce HIV acquisition have important wider benefits. For example, needle and syringe programmes are effective at preventing HIV, but also prevent hepatitis C infections, and reduce the risks of bacterial abscesses and endocarditis. For another example, provision of school-based comprehensive sexuality education in regions with low HIV prevalence will lead to many substantive benefits for both girls and boys and will also reduce the chances of HIV acquisition still further. Costs of such services need to be appropriately attributed in developing economic arguments for HIV prevention.

### Choice of HIV prevention methods

The range of prevention options means that individuals now have more ability to exercise their autonomy and agency. Individuals need an accurate perception of their risk of exposure as well as agency to adopt the best available solutions for themselves and their partners.

The wide range of options for how to avoid infection with HIV includes behavioural as well as biomedical approaches. Choosing partners, including by HIV status and viral suppression status, choosing how and when sex happens, choosing condoms and lube, choosing PrEP, choosing to access sexual and reproductive health services (including antenatal care for pregnant women), choosing clean needles and syringes for injection, and choosing PEP. Individuals’ choices will vary over time and with different partners. It is appropriate that some approaches will be used on some occasions and not others.

For example, the gay men’s health surveys in Australia demonstrate increasing levels of overall protection, while rates of condom use decline and uptake of other prevention options (notably PrEP) increase [25].

### HIV prevention that is appropriate to individuals’ circumstances

Available prevention approaches should be appropriate to each individual’s circumstances and vulnerability to HIV. Specifics prevention approaches are developed with different populations in mind. Examples include services for prevention of mother to child transmission, harm reduction approaches for people who inject drugs, and topical PrEP products adapted for rectal or vaginal sex. Appropriate advertising, packaging, and presentation of products for different population segments using human-centred design and other techniques are being learned from private sector marketing methods [26].

An important element of appropriateness is the timing and frequency of use. Event-driven PrEP, where 2 pills are taken before sex and 2 further pills taken 24 and 48 hours later has been shown to be highly effective at preventing HIV acquisition through anal sex [11]. It is therefore particularly appropriate for those men who have sex with men less frequently and that is not protected either by a condom or by accurate knowledge of a partner’s serostatus or viral load status. Similar considerations may also be important for PrEP for vaginal sex. Many people who start oral PrEP do not remain on it after several months [27]. In some, the reason for stopping PrEP may be related to a reduction in risk of HIV acquisition for behavioural or partnership-related reasons [28]. Some people can predict periods when they will be at more exposed to HIV and so choose to start and subsequently stop PrEP [29]. Such individuals may or may not want a product that lasts many months at a time, such as an implant or a long-acting injection. Periods of exposure also relate to the cost of the approach. For those who know that they remain exposed, a model of widely available but intermittent PrEP usage might expand the cost-efficacy envelope considerably [30].

### Prioritised approaches to HIV prevention

As new tools become available, it will be important to ensure that those who need them most are effectively using them. Low coverage of HIV services among key populations often stems from societal and legal barriers that create environments where people living with HIV and people who are exposed to HIV infection may not feel safe to utilise health services. In settings where there is less homophobia and men who have sex with men can access sexual health services including HIV and sexually transmitted infection testing and treatment and PrEP, the number of new infections has fallen substantially [31,32]. Yet, in those areas of the US with the highest incidence of HIV among MSM, the uptake of PrEP has been lower [33].

One of the most striking features of the epidemiology of HIV is the heterogeneous distribution across geographies [34]. More sophisticated mathematical models are providing better estimates of incidence at subnational levels and highlighting the huge variation in incidence within countries and across different ages and genders [16,35]. The wide range of incidence in different districts in countries in east, central, and southern Africa is demonstrated in the S1 Text.

A consequence of this skewed distribution of HIV incidence, is that the costs of commodities needed to prevent each new HIV infection with any biomedical approach rises steeply if prevention is not prioritised towards those most likely to benefit [36]. A major challenge remains how to achieve these cost efficiencies. In settings where background HIV incidence is already high, simple self-selection may be sufficient [37], although the evidence is mixed [38], but in settings where the burden of HIV is lower, some preliminary focusing of efforts is likely to be needed. Geography, age, and gender allow a crude stratification, which can be enhanced, e.g., by self-reports of number of sexual partners or history of sexually transmitted infections. Such screening approaches may be counter-productive if not delivered in a socially acceptable way. Nonetheless, even with inevitable under-reporting, data from recent surveys do show much higher measured HIV incidence in those who do report multiple partners or recent sexually transmitted infections [39].

HIV acquisition within key populations also varies greatly depending on individual behaviours and the spatial variation in epidemiology. Again, self-selection may be sufficient, but is likely to miss many who might benefit, and conversely to provide services to some who probably have a lower chance of acquiring infection [40]. For example, sex workers in urban areas in eastern and southern Africa are at higher risk than those working in low prevalence areas, including those working in rural areas [41]. For another example, the incidence of HIV among men having sex with men who attended sexual health services in Barcelona more than once (which allowed measurement of HIV incidence) showed large variation among those who reported different behaviours [42].

To maximise the utility and impact of new prevention technologies, their costs will need to fall substantially in low- and middle-income countries. If such technologies (including long-acting ARV products, topical rings, and eventually broadly neutralising antibody combinations and vaccines) are to be offered to people whose chance of acquiring HIV is less than 1% per year (which is already a considerable risk, see S1 Text), then more than 100 people will have to receive the product for a year to prevent each new HIV infection directly. The cost of commodities for such approaches may therefore be very large to have an important impact on the burden of new infections in “generalised” epidemics [43]. For vaccines, the durability of protection will be a key economic consideration.

In many key populations, incidence rates are higher, and the new technologies may therefore have a greater impact at more affordable costs. More intermittent use of PrEP, focused on shorter periods of known or anticipated risk, would improve efficiency but will be harder to implement for long-acting PrEP options [30]. Post exposure prophylaxis for HIV could be more widely used, in an approach analogous to emergency contraception, for those people who are not regularly exposed to HIV. This could also cut the costs of ARV-based HIV prevention for some populations. New single dose post-exposure prophylaxis could therefore be a significant advance for many people [44].

Prioritisation does not preclude choice and appropriateness of HIV prevention nor is it a rigid process, but rather a means to allow programme staff and policy makers to maximise the equity, utility, and impact of prevention services. Furthermore, as HIV incidence continues to decline, the importance of prioritisation will increase [45].

### Person-centred prevention

The importance of centering prevention on the person includes the elements of choice and appropriateness above. However, individuals are often not the architect of their own destiny, and those left behind are often from the most marginalised and discriminated communities.

Repeated social science studies demonstrate that for many people at risk of HIV, particularly young people and those from marginalised or criminalised key populations, existing health services that purport to offer HIV treatment and prevention services are not places that welcome the very people who need those services most [46]. Person-centred prevention links to the broader discussions of societal and legal barriers [47] and ensuring that services are community led [23] as well as improving the delivery of services.

### Combination HIV prevention

The 2025 targets on HIV prevention allow for a combination of effective prevention options for people at risk of HIV. The framework acknowledges that a different combination of interventions may be needed for a particular individual sex or injection act. Combination prevention emphasises the need for behavioural and structural intervention alongside the biomedical approaches (which themselves require behavioural intervention to maximise adherence and thus efficacy).

Combined services also include the provision of integrated service delivery, with the goal of providing tailored, co-located, or well-coordinated services that are optimally convenient, seamless, and easy to navigate [48].

Multipurpose technologies include methods that are effective not only in HIV prevention but also serve other key purposes, such as contraception or preventing other sexually transmitted infections [49]. As discussed above, the economic evaluation and costing of such methods needs to apportion cost and benefits appropriately.

### Specific HIV prevention targets

In order to model global estimates or the impact and resource needs for the HIV response over the next years, specific prevention targets have been proposed [3,50] and are described more fully in the S1 Text. However, these should be seen as global targets for what needs to be reached if we are to achieve the goal for ending AIDS by 2030.

The process to translate these targets into impact is described elsewhere and involves interpolation and imputation for missing data [50]. The specific targets aim to promote the principles encapsulated in the wording of the overarching target. They should allow national planners to define nationally appropriate targets for their own priorities and populations.

### Policy, implementation, and next steps

The new targets adopted for the global HIV response are ambitious and innovative. They outline principles and priorities for national programmes to change the course of their HIV programme to maximise the chances of reaching the global sustainable development goals by 2030.

These targets have major implications for programmes. The overarching target moves away from linear cascades for specific tools, focusing instead on the proportion of people with access and who are able to choose to use appropriate prevention methods. By setting ambitious targets for all HIV services, the divisions between treatment and prevention are reduced; the major contribution of viral load suppression to incidence reduction is acknowledged; and primary prevention is given more visibility and priority. Prioritisation of prevention requires greater use of subnational and local data sources and models and implies better estimation of the size of different key populations as well as stronger monitoring and evaluation of delivery of interventions. Enhanced surveillance systems will also detect new localised outbreaks of HIV in real time allowing appropriate streamlined course corrections to be made.

The HIV service targets, including these prevention principles and targets, are an integral part of the whole package of targets within the global HIV strategy. Increasingly, HIV services will need to be integrated within the larger health sector and within the context of universal health coverage. These prevention efforts will not succeed unless there are strong links with human rights and clear intersections with efforts to reduce structural and societal barriers [47].

The new framing of prevention emphasising inequalities, agency, and local data for decision-making will prevent new infections, reduce deaths, and stigma and discrimination and as a core element of the new Global HIV Strategy can restore the trajectory to end AIDS as a public health threat by 2030. Furthermore, the focus on person-centred approaches has already reinforced resilience in the face of the Coronavirus Disease 2019 (COVID-19) pandemic [23].

The prevention principles and targets offer an agenda for action, the next challenge is to ensure that sufficient resources, human, financial, and advocacy are committed to deliver for the future.

## Supporting information

S1 Text Examples of heterogeneity of modelled HIV incidence across 5 high burden countries. Criteria, thresholds, and levels used in model of impact and resource needs.
(DOCX)
