# Correction: HIV prevention for the next decade: Appropriate, person-centred, prioritised, effective, combination prevention

**DOI:** 10.1371/journal.pmed.1004131

**Published:** 2022-11-04

**Authors:** The PLOS Medicine Staff

S1 Text contains an incorrect version of Fig 1C. Please see the correct S1 Text file below. The publisher apologizes for the error.

## Supporting information

S1 Text Examples of heterogeneity of modelled HIV incidence across 5 high burden countries. Criteria, thresholds, and levels used in model of impact and resource needs.
(PDF)
