# Supplementary material for: HIV prevention for the next decade: Appropriate, person-centred, prioritised, effective, combination prevention
Source: PLoS Med. 2022 Sep 26;19(9):e1004102. doi: 10.1371/journal.pmed.1004102 (PMC9550175; doi:10.1371/journal.pmed.1004102)
Supplement: S1 Text — Criteria, thresholds, and levels used in model of impact and resource needs. (DOCX) [file pmed.1004102.s001.docx]

## **Policy Forum for PLoS Collection on HIV Targets**

**HIV prevention for the next decade: Appropriate, Person-centred, Prioritised, Effective, Combination Prevention**

Supplementary table and figures.

One of the most striking features of the epidemiology of HIV is the heterogeneous distribution across geographies [1]. More sophisticated mathematical models are providing better estimates of incidence at sub-national levels and highlighting the huge variation in incidence within countries and across different ages and genders. Table 1 shows the highest, lowest and median estimated incidence per 100 person-years among women and men aged 15-24 in districts of five countries in East, Central and Southern Africa [2]. Figure 1 shows the range of incidence among women aged 15-24 across all districts in the same countries with the districts from Table 1 highlighted. In each case there is a several fold difference between the incidence in different sub-national administrative areas. What is also striking, is that several recent clinical trials in these same countries have recruited young women in whom the incidence measured in the control or placebo arms was considerably higher than these averages [3,4,5].

Table 1: Examples of sub-national areas in East and Southern Africa with diverse HIV epidemics by age and sex: 2020

| Country | Sub-national area | HIV Incidence(and 95% credible interval)  per 100 person-years among young women 15-24 | | HIV Incidence (and 95% credible interval) per 100 person-years among young men 15-24 | |
| --- | --- | --- | --- | --- | --- |
| Mozambique (High) | Mocubela | 2.35 | 1.57-3.34 | 0.71 | 0.47-1.01 |
| Mozambique (Median) | Vilankulu | 0.79 | 0.54-1.11 | 0.25 | 0.17-0.35 |
| Mozambique (Low) | Tsangano | 0.05 | 0.03-0.09 | 0.02 | 0.01-0.03 |
| South Africa (High) | Oliver Tambo DM | 1.99 | 1.26-3.07 | 0.58 | 0.31-0.98 |
| South Africa (Median) | Ekhurhuleni MM | 1.13 | 0.71-1.18 | 0.23 | 0.12-0.41 |
| South Africa (Low) | Central Karoo DM | 0.47 | 0.26-0.77 | 0.13 | 0.06-0.25 |
| Uganda (High) | Kalangala | 0.72 | 0.52-1.01 | 0.25 | 0.18-0.35 |
| Uganda (Median) | Mbale | 0.21 | 0.16-0.28 | 0.06 | 0.04-0.08 |
| Uganda (Low) | Yumbe | 0.04 | 0.03-0.07 | 0.01 | 0.01-0.02 |
| Kenya (High) | Kisumu | 0.69 | 0.58-0.81 | 0.24 | 0.2-0.28 |
| Kenya (Median) | Machakos | 0.12 | 0.09-0.15 | 0.02 | 0.02-0.03 |
| Kenya (Low) | Mandera | 0 | 0-0.01 | 0 | 0 |
| Zimbabwe (High) | Bulilima | 0.93 | 0.76-1.14 | 0.31 | 0.25-0.37 |
| Zimbabwe (Median) | Chitungwiza | 0.42 | 0.34-0.53 | 0.12 | 0.09-0.15 |
| Zimbabwe (Low) | Centenary | 0.17 | 0.13-0.22 | 0.07 | 0.05-0.09 |

Source: UNAIDS Epidemiological Estimates 2021. https://naomi-spectrum.unaids.org/

Figure 1.a-e

Estimated incidence of HIV per 100 person years among women aged 15-24 in sub-national areas across five African countries, ranked from highest to lowest.


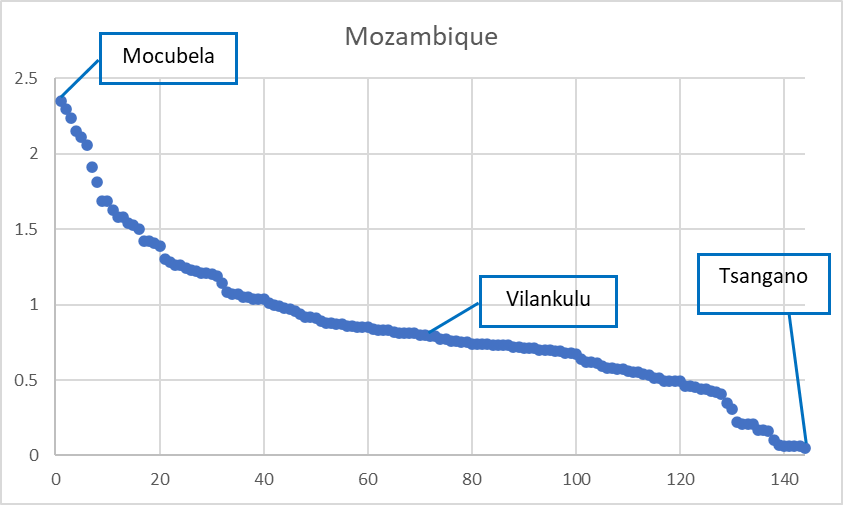


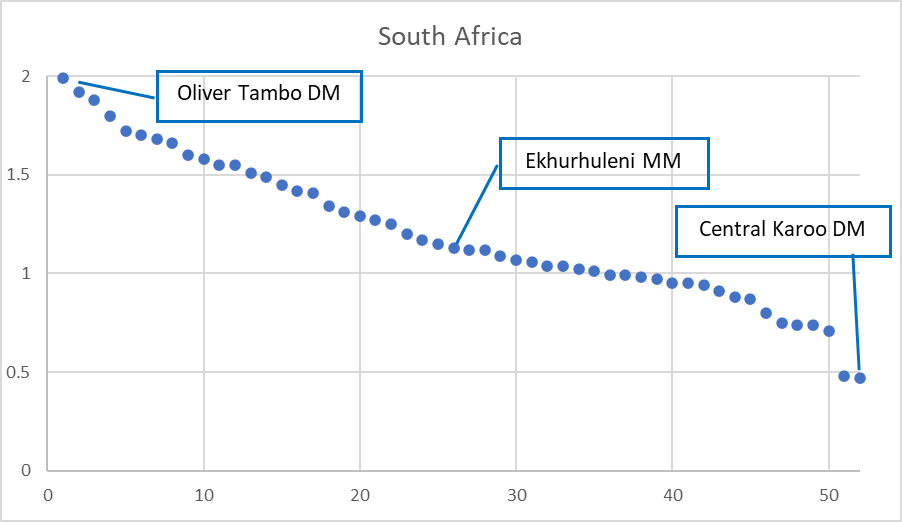


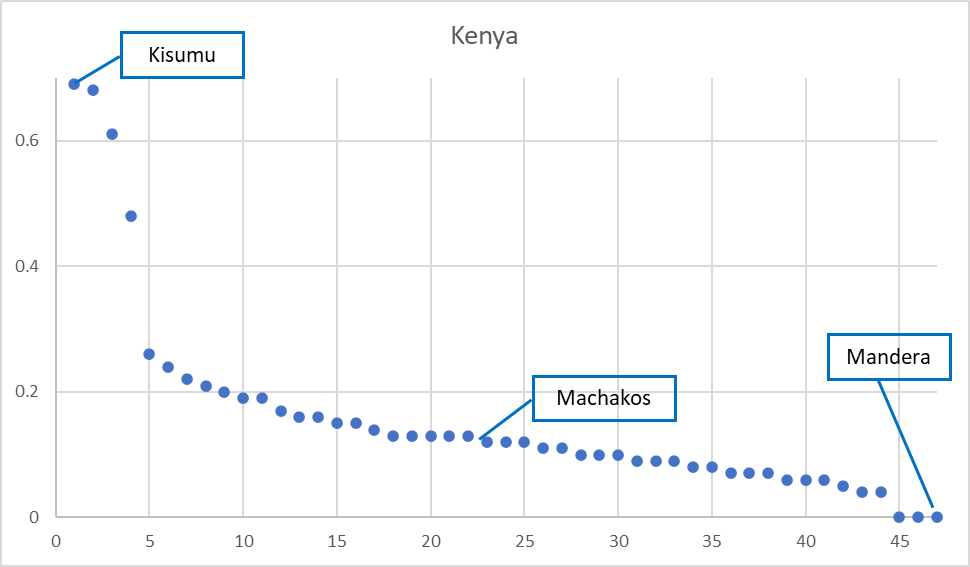


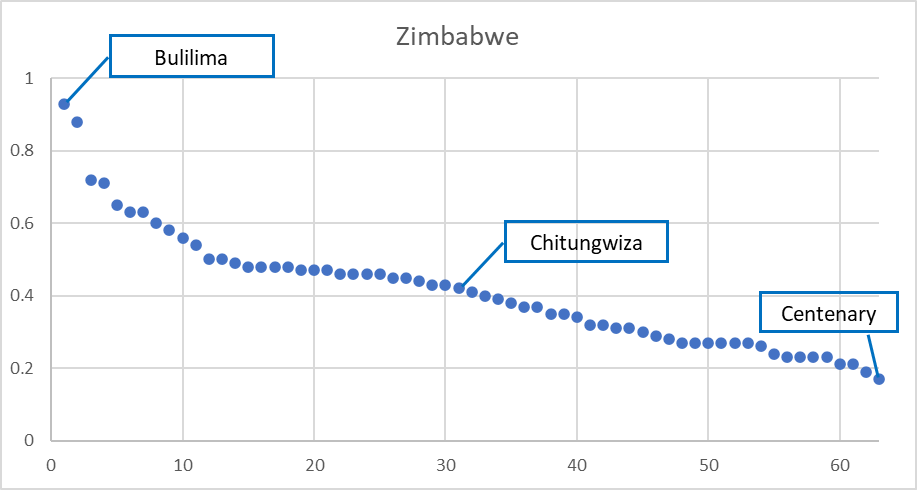


https://naomi-spectrum.unaids.org/

## Specific HIV prevention targets.

In order to model global estimates or the impact and resource needs for the HIV response over the next years, specific prevention targets have been proposed and are shown in figure 2 [6].

However, these should be seen as global targets for what needs to be reached if their impact is to achieve the global targets for 2030. Prioritised prevention can be stratified according to the level of HIV in different populations as follows: A geographical focus for sex workers and prisoners (based on the background national adult prevalence); a behavioural focus for gay/MSM, transgender people and people who inject drugs (based on survey data and models of risk); a geographical and behavioural approach for AGYW, ABYM, and adults >25 (based on models of subnational incidence and reported behavioural risk) [7,8].

The process to translate these targets into impact is described elsewhere, and involves interpolation and imputation for missing data [7]. The specific targets aim to promote the principles encapsulated in the wording of the overarching target. They should allow national planners to define nationally appropriate targets for their own priorities and populations.


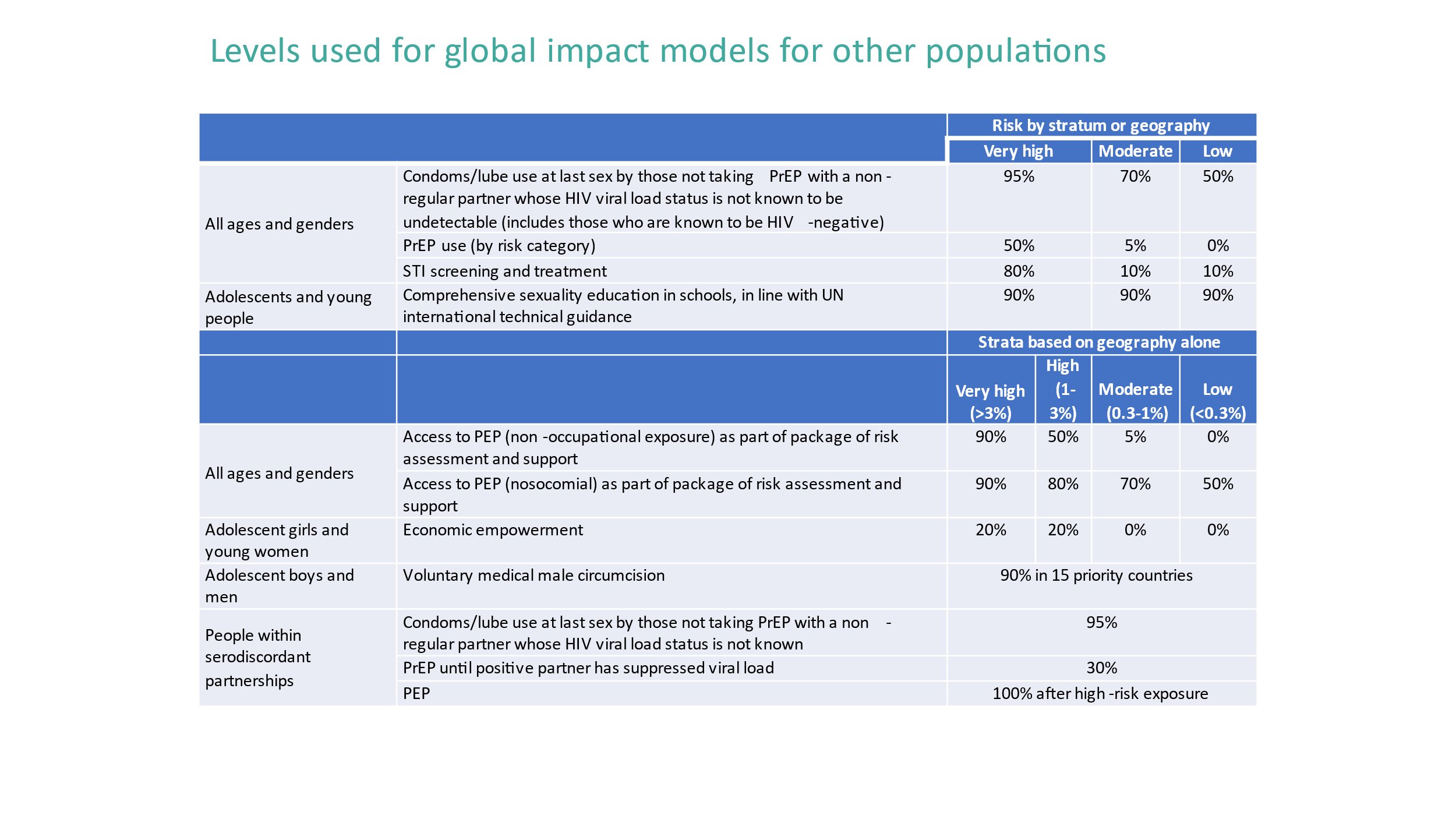

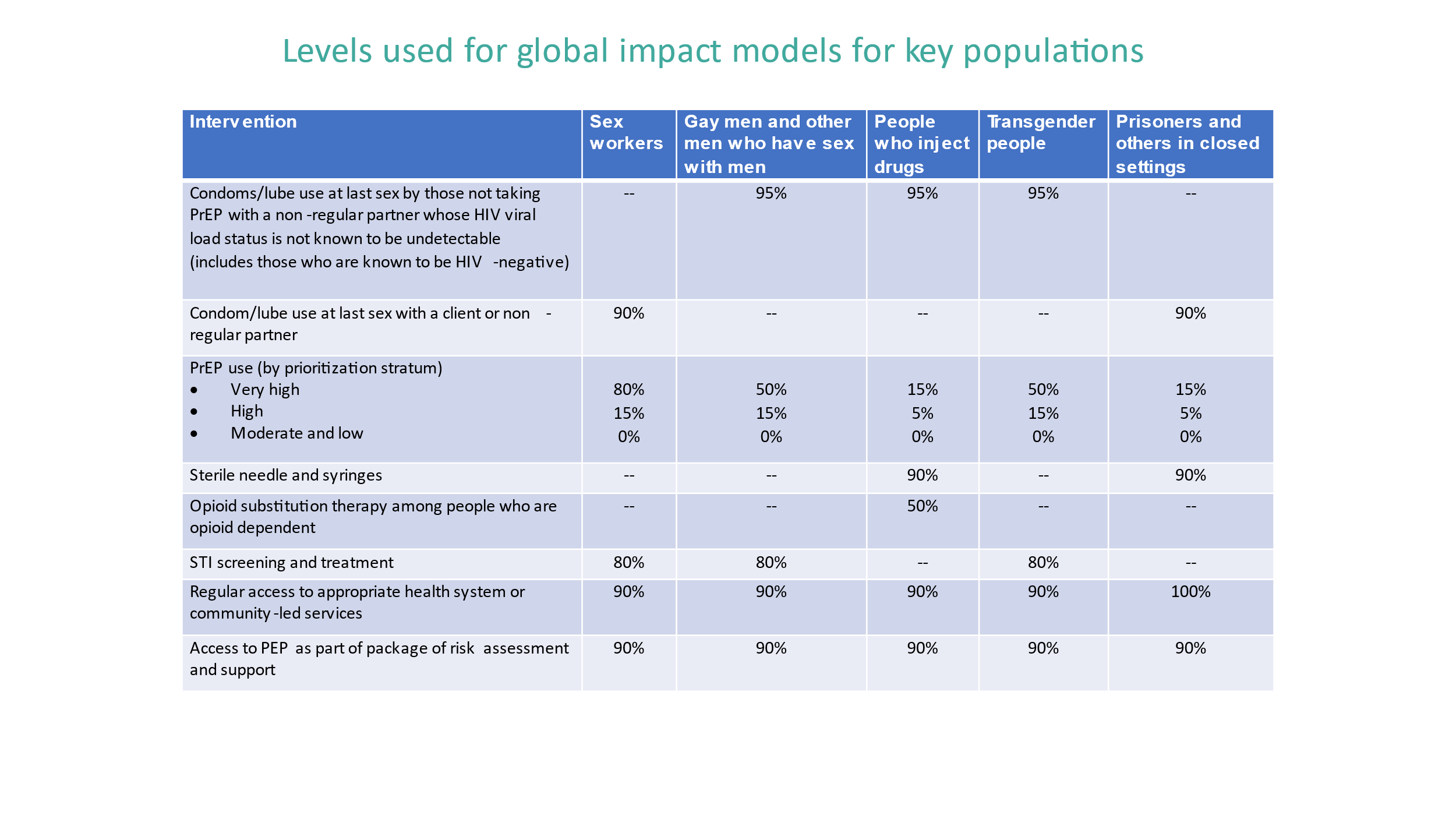

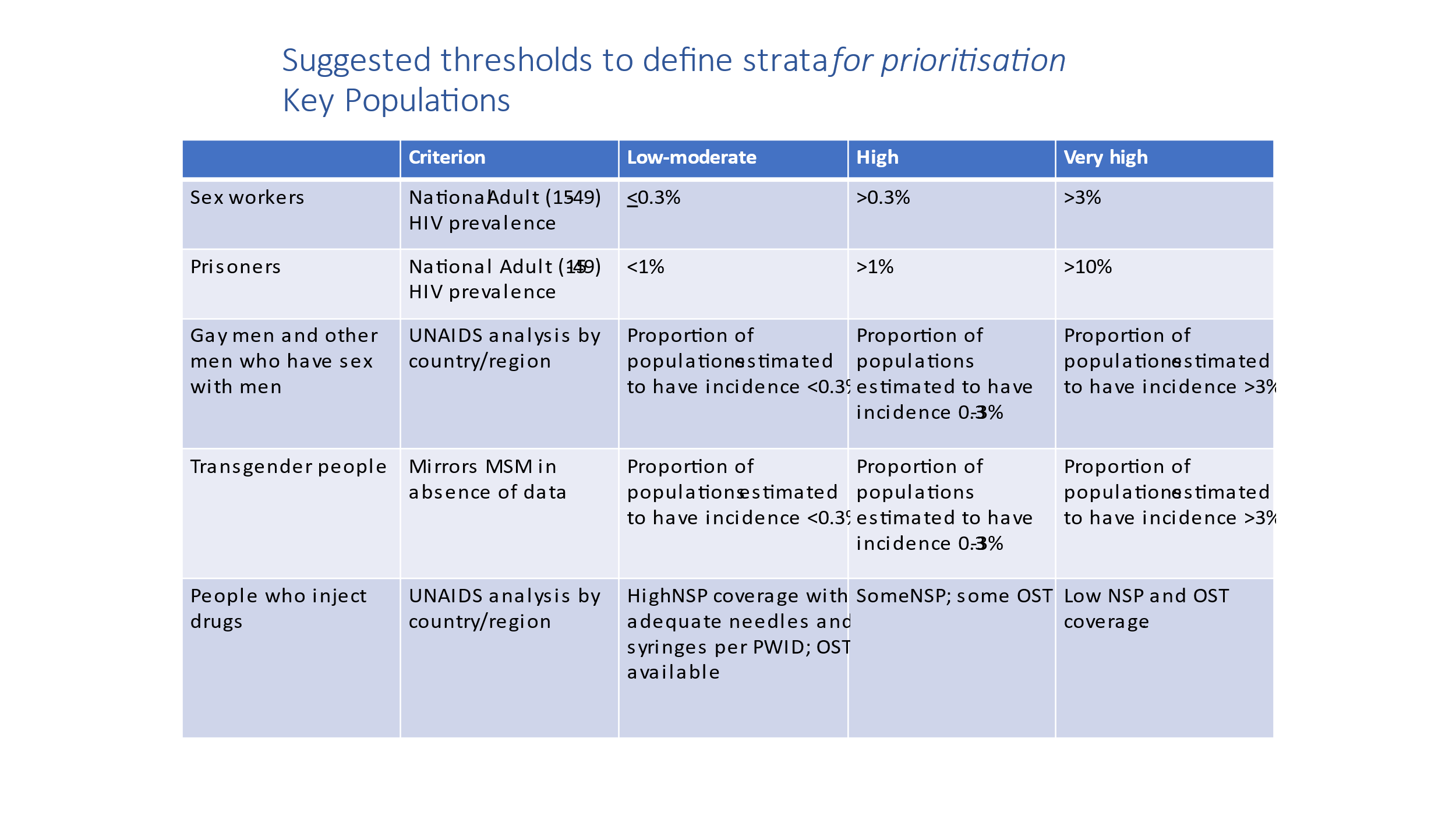

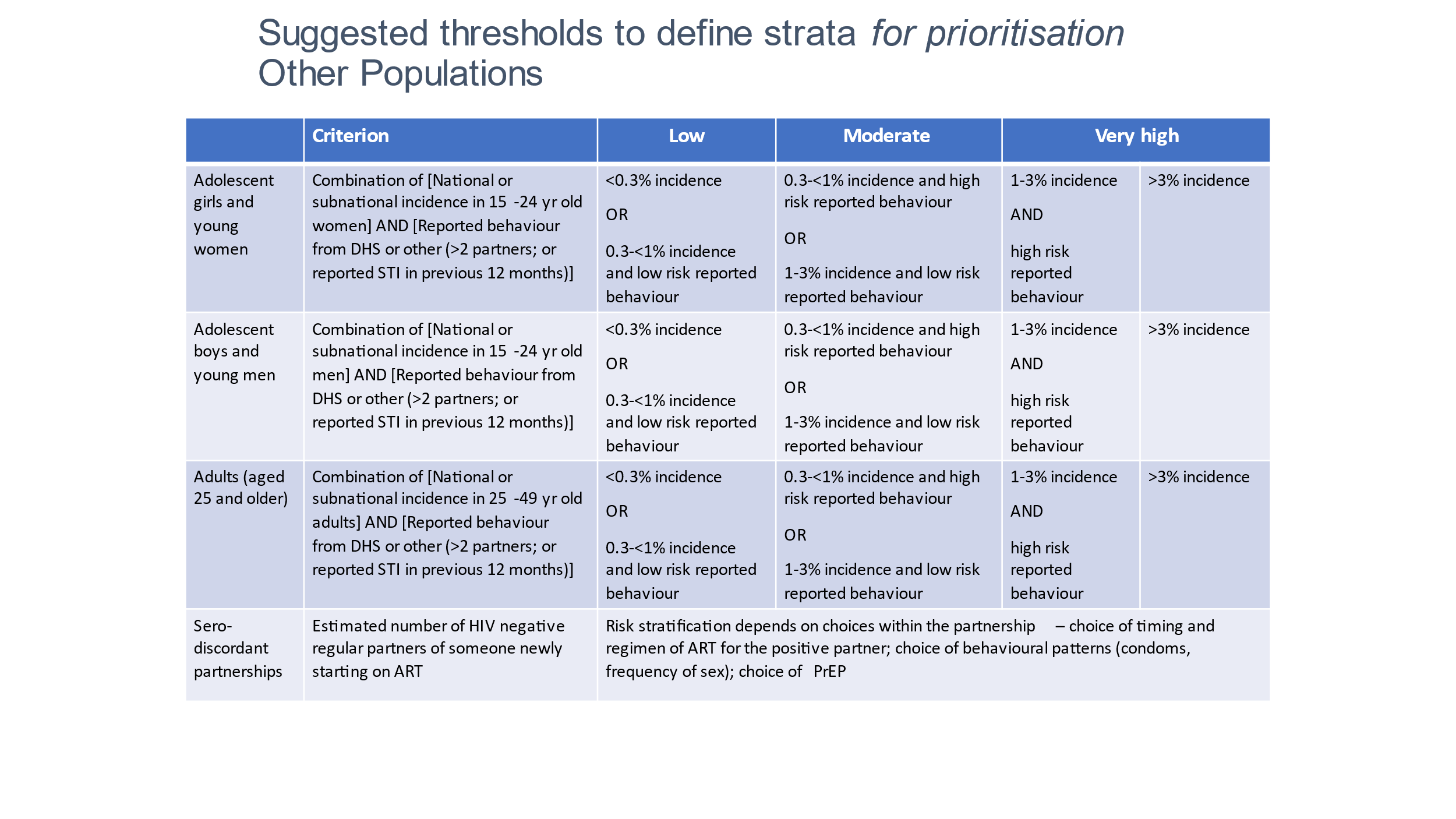
Figure 2: Criteria for stratification of countries or subnational areas; thresholds for stratification and targets for each stratum. (See UNAIDS Strategy and Targets documentation for fuller description) [9]

Note: Incidence is measured or modelled for each 5yr age and sex cohort in “other populations”

Abbreviations in figure 2:

MSM: Gay men and other men who have sex with men

NSP: Needle and Syringe Programme

OST: Opioid Substitution Therapy

PWID: People who inject drugs

PrEP: Pre-exposure prophylaxis

PEP: Post-exposure prophylaxis

References

1. Coburn BJ, Okano JT, Blower S. Using geospatial mapping to design HIV elimination strategies for sub-Saharan Africa. Sci Transl Med. 2017 Mar 29;9(383):eaag0019. doi: 10.1126/scitranslmed.aag0019. PMID: 28356504; PMCID: PMC5734867.
2. UNAIDS <https://naomi-spectrum.unaids.org/> (accessed 17jul2022)
3. Nel A, van Niekerk N, Kapiga S, Bekker LG, Gama C, Gill K, et al. Ring Study Team. Safety and Efficacy of a Dapivirine Vaginal Ring for HIV Prevention in Women. N Engl J Med. 2016 Dec 1;375(22):2133-2143. doi: 10.1056/NEJMoa1602046. PMID: 27959766.
4. Baeten JM, Palanee-Phillips T, Brown ER, Schwartz K, Soto-Torres LE, Govender V, et al. MTN-020–ASPIRE Study Team. Use of a Vaginal Ring Containing Dapivirine for HIV-1 Prevention in Women. N Engl J Med. 2016 Dec 1;375(22):2121-2132. doi: 10.1056/NEJMoa1506110. Epub 2016 Feb 22. PMID: 26900902; PMCID: PMC4993693.
5. Evidence for Contraceptive Options and HIV Outcomes (ECHO) Trial Consortium. HIV incidence among women using intramuscular depot medroxyprogesterone acetate, a copper intrauterine device, or a levonorgestrel implant for contraception: a randomised, multicentre, open-label trial. Lancet. 2019 Jul 27;394(10195):303-313. doi: 10.1016/S0140-6736(19)31288-7. Epub 2019 Jun 13. Erratum in: Lancet. 2019 Jul 27;394(10195):302. PMID: 31204114; PMCID: PMC6675739.
6. <https://www.jnj.com/johnson-johnson-and-global-partners-announce-results-from-phase-2b-imbokodo-hiv-vaccine-clinical-trial-in-young-women-in-sub-saharan-africa> (accessed 17jul2022)
7. Stover J, Glaubius R, Teng Y, Kelly S, Brown T, Hallett TB, et al. Modeling the epidemiological impact of the UNAIDS 2025 targets to end AIDS as a public health threat by 2030. PLoS Med. 2021 Oct 18;18(10):e1003831. doi: 10.1371/journal.pmed.1003831. PMID: 34662333; PMCID: PMC8559943
8. UNAIDS Global AIDS Strategy 2021-26 <https://www.unaids.org/en/resources/documents/2021/2021-2026-global-AIDS-strategy> (accessed 17jul2022)
9. UNAIDS 2025 Targets page <https://www.unaids.org/en/topics/2025_target_setting> (accessed 17jul2022)
